# Effects of the DL76 Antagonist/Inverse Agonist of Histamine H_3_ Receptors on Experimental Periodontitis in Rats: Morphological Studies

**DOI:** 10.3390/ph17060792

**Published:** 2024-06-17

**Authors:** Mariusz Geremek, Bogna Drozdzowska, Dorota Łażewska, Katarzyna Kieć-Kononowicz, Jerzy Jochem

**Affiliations:** 1 Department of Public Health, Faculty of Public Health in Bytom, Medical University of Silesia in Katowice, 40-055 Katowice, Poland; 2 Department of Pathomorphology, Faculty of Medical Sciences in Zabrze, Medical University of Silesia in Katowice, 40-055 Katowice, Poland; 3 Department of Technology and Biotechnology of Drugs, Faculty of Pharmacy, Jagiellonian University Medical College, 9 Medyczna St, 30-688 Kraków, Poland; 4 Department of Physiology, Faculty of Medical Sciences in Zabrze, Medical University of Silesia in Katowice, 40-055 Katowice, Poland

**Keywords:** periodontitis, H_3_ receptor antagonist/inverse agonist, RANKL/RANK/OPG

## Abstract

Background: Periodontitis preceded by gingivitis is the most common form of periodontal disease and occurs due to the interaction of microorganisms present in the complex bacterial aggregates of dental plaque biofilm and their metabolism products with periodontal tissues. Histamine is a heterocyclic biogenic amine acting via four types of receptors. Histamine H_3_ receptors act as presynaptic auto/heteroreceptors to regulate the release of histamine and other neurotransmitters. Aim: Since the nervous system is able to regulate the progression of the inflammatory process and bone metabolism, the aim of this study was to investigate the effects of DL76, which acts as an antagonist/inverse agonist of H_3_ receptors, on the course of experimental periodontitis. Materials and methods: This study was conducted in 24 mature male Wistar rats weighing 245–360 g, aged 6–8 weeks. A silk ligature was placed on the second maxillary molar of the right maxilla under general anesthesia. From the day of ligating, DL76 and 0.9% NaCl solutions were administered subcutaneously for 28 days in the experimental and control groups, respectively. After the experiment, histopathological, immunohistochemical and radiological examinations were performed. Results: Ligation led to the development of the inflammatory process with lymphocytic infiltration, increased epithelial RANKL and OPG expression as well as bone resorption. DL76 evoked a reduction in (1) lymphocytic infiltration, (2) RANKL and OPG expression as well as (3) bone resorption since the medians of the mesial and distal interdental spaces in the molars with induced periodontitis were 3.56-fold and 10-fold lower compared to the corresponding values in saline-treated animals with periodontitis. Conclusion: DL76 is able to inhibit the progression of experimental periodontitis in rats, as demonstrated by a reduction in the inflammatory cell infiltration, a decrease in the RANKL/RANK OPG pathway expression and a reduction in the alveolar bone resorption.

## 1. Introduction

Periodontitis is characterized by the degradation of the supporting apparatus of the tooth (gingival tissue, alveolar bone, periodontal ligament and cementum) [1]. Periodontitis preceded by gingivitis is the sixth most prevalent chronic disease globally, with severe symptoms occurring in about 11.2% of the population [2]. It can lead to tooth loss and is one of the main health challenges regardless of geographic location [3].

The etiology of periodontal diseases is complex. They are caused by the interaction of Gram-negative microorganisms present in the complex bacterial aggregates of dental plaque biofilm and their metabolism products with periodontal tissues [4,5,6,7]. This process is modified by the host inflammatory response [8], which determines the advance, severity and progression of the disease as well as the effects of the therapy [1]. In addition, modifiable and non-modifiable risk factors are able to affect the progression of periodontitis. The modifiable risk factors include dental plaque and/or tartar control, orthodontic disorders, low-quality dentures and dental fillings that project beyond the margin of a tooth as well as lifestyle risk factors (i.e., tobacco smoking, excessive alcohol consumption, smoking, improper nutrition resulting in overweight and obesity) [1,2]. The non-modifiable risk factors include age-related physiological changes, gender, ethnicity, genetic factors, diabetes, AIDS and Crohn’s disease [2,3].

At the beginning of bacterial colonization, an increased number of neutrophils can be found in the connective tissue, together with other cells of the immunological system (i.e., macrophages, mast cells) [9]. As the inflammation develops, macrophages and both lymphocytes T and B can be observed and the collagenolytic activity increases [9]. If the inflammatory process advances, the loss of supporting connective tissue (attachment loss) and alveolar bone loss due to the activation of osteoclastic activity can be diagnosed [10]. Finally, the formation of a periodontal pocket occurs [10,11]. Different intrabonal effects appear as a result of the osteoclast-induced periodontal destruction of bone [12].

Osteoclasts are part of the mononuclear phagocyte system. They are derived from mature monocytes and macrophages [13]. Alveolar bone loss, which results from osteoclast activity, depends on the RANKL/RANK/OPG pathway [13].

The transmembrane receptor activator of nuclear factor-κB (RANK) is expressed in osteoclasts. The receptor activator of nuclear factor-κB ligand (RANKL) determines osteoclast activity. RANKL, which is discharged by osteoblasts, binds to RANK and is necessary for osteoclasts differentiation [13]. Osteoprotegerin (OPG), which is secreted by stromal cells, is a decoy receptor for RANKL and, in that way, is able to regulate the activity of the RANK signaling pathway by competing for RANKL [14]. It inhibits osteoclastogenesis by impeding RANKL/RANK binding [14,15,16]. An increased RANKL/OPG ratio characterizes the periodontal tissues during the inflammatory process [15,16].

Histamine 2-(1H-imidazol-4-yl)ethylamine) is a heterocyclic biogenic amine, a product of decarboxylation of L-histidine [17]. It exerts biological effects by acting via four types of receptors H_1_–H_4_ [17]. The release of histamine in gingival tissues in the first phase of the inflammatory process is induced by bacterial antigens [17,18]. Activation of the H_1_ and H_2_ receptors located on gingival fibroblasts, as well as the H_4_ receptors on neutrophils and T-lymphocytes, modulates the level of secretion of pro-inflammatory cytokines and the increase in osteoclastogenesis in the alveolar bone [19]. Histamine influences osteoclastic resorption of bone as it modifies osteoclast precursor recruitment and differentiation and appears to be a crucial factor in the osteoclast and osteoblast interaction [20]. In addition, the course of the inflammatory reaction is modulated by histamine, which regulates bone metabolism [21]. The mechanism is associated with the osteoblast expression of H_1_ and H_2_ receptors and the histamine-induced increase in RANKL release [20,21].

Histamine H_3_ receptors were first described in 1983 [22]. As presynaptic receptors, they control the synthesis and release of neurotransmitters (i.e., histamine, dopamine, serotonin and acetylcholine) in the central and peripheral nervous system, especially in the cerebral cortex, basal ganglia, hippocampus and pre- and postganglionic neurons of the sympathetic nervous system [23].

The nervous system is able to regulate the progression of the inflammatory process [24] as well as bone metabolism [25]. Denervation significantly exacerbates ligature-induced rat periodontitis, which is associated with an increased RANKL/OPG ratio, nuclear factor-κB signaling pathway activation and consecutive production of IL-1β and TNF-α as well as an increased number of osteoclasts [26]. Therefore, the aim of this study was to assess the possible influence of the H_3_ receptor antagonist/inverse agonist DL76 on experimentally induced periodontitis by estimating the histological and anatomical changes, as well as the RANKL/RANK/OPG pathway activity, in the periodontal tissues.

## 2. Results

### 2.1. Histopathological Results

**Figure 1:**
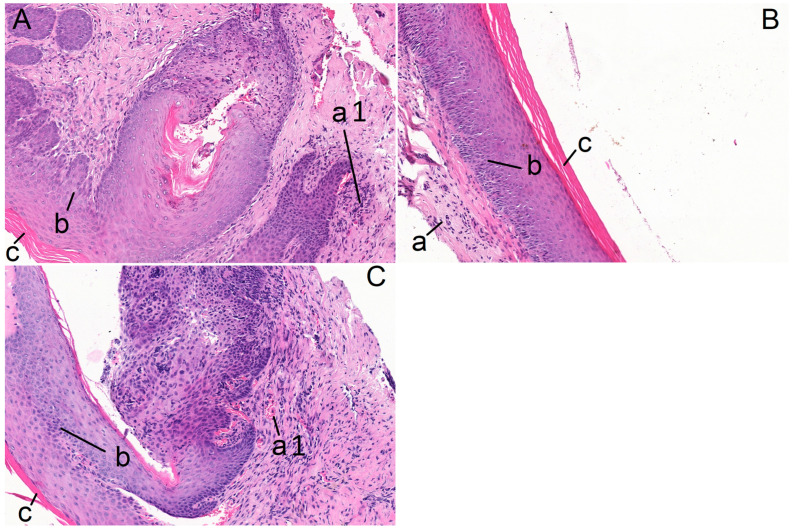
Histopathological examination of the gingival tissue of the alveolar process in the S_R_ (**A**), S_L_ (**B**) and DL_R_ (**C**) specimens; a—lamina propria, a1—lymphocytic infiltration in the lamina propria, b—stratified squamous epithelium, c—keratinized layer (40×).

In the saline-treated control group, symptoms of inflammation with lymphocytic infiltration were found in all the samples of molar mucosa in the S_R_ samples (*n* = 6), whereas there were no signs of inflammation (0% of cases) in the S_L_ samples (*n* = 6). Symptoms of inflammation were found in 50% of the DL_R_ specimens (*n* = 6), while there were no signs of inflammation (0% of cases) in the DL_L_ samples (*n* = 6) (Figure 1).

### 2.2. IHC Results

There were no statistically significant differences in the epithelial RANK expression between the S_R_ and S_L_ samples, with the median values being 72.5% and 67.5%, respectively. Similarly, there were no differences between the DL_R_ (70%) and DL_L_ (65%) in terms of the value of RANK (Table 1).

The median value of the epithelial RANKL expression in the S_R_ samples was significantly higher compared to S_L_ (*p* = 0.048), DL_L_ (*p* = 0.02) and DL_R_ (*p* = 0.048).

The epithelial OPG expression in the S_R_ samples was significantly higher than in the S_L_ (*p* = 0.02) and DL_L_ (*p* = 0.02) samples (Table 1).

Subsequently, it was checked whether there was any statistically significant relationship between the variables in the studied groups. A statistically significant correlation was found in the following variables: RANK in the stroma, RANKL in the epithelium, OPG in the epithelium and OPG in the stroma. The results indicate that in the S_L_, S_R_ and DL_R_ samples, low RANK expression in the stroma was dominant, while in the DL_L_ samples, the lack of expression was dominant (Table 2).

The lack of RANKL expression in the epithelium was dominant in the S_L_, DL_R_ and DL_L_ samples, and low expression in the S_R_ samples (Table 2).

No expression of OPG in the epithelium was dominant in the S_L_, DL_R_ and DL_L_ samples, while low expression was dominant in the S_R_ samples (Table 2).

A lack of stromal OPG expression was found in the S_L_, DL_R_ and DL_L_ samples. In the S_R_ samples, 50% of the results referred to a lack of expression, whereas 50% of the results indicated the low expression of OPG in the stroma (Table 2).

### 2.3. Radiological Results

**Figure 2:**
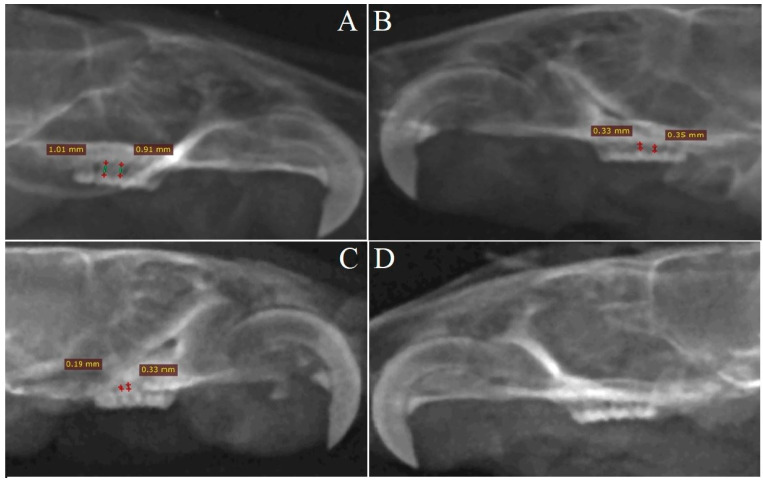
Alveolar bone loss in the mesial and distal interdental spaces (in millimeters, mm) in the studied groups ((**A**)—S_R_, (**B**)—S_L_, (**C**)—DL_R_, (**D**)—DL_L_).

The alveolar bone loss in the mesial interdental spaces in the S_R_ maxillas was significantly higher compared to the mesial interdental spaces in the S_L_ maxillas (*p* = 0.005) (Figure 2A,B).

The value of the alveolar bone loss in the S_R_ mesial interdental spaces was significantly higher compared to DL_R_ (*p* < 0.001) and DL_L_ (*p* < 0.001) (Table 3). There were no differences concerning the alveolar bone loss in the mesial interdental spaces between DL_L_ and DL_R_ (Figure 2C,D). There were no statistically significant differences concerning the alveolar bone loss in the mesial interdental spaces between DL_R_ and S_L_ (*p* = 0.08) (Table 3).

The alveolar bone loss in the S_R_ distal interdental spaces was significantly higher compared to S_L_ (*p* = 0.006) (Table 3).

The alveolar bone loss in the S_R_ distal interdental spaces was significantly higher compared to DL_L_ (*p* = 0.001) and DL_R_ (*p* < 0.001) (Table 3), whereas there were no differences between DL_L_ and DL_R_ (Table 3).

## 3. Discussion

In the present study, we demonstrated for the first time DL76-mediated inhibition of the inflammatory process and the maxillary alveolar bone resorption in a rat model of periodontitis. We also described the influence of DL76 on the RANKL/RANK/OPG pathway in the course of experimental periodontitis.

Periodontitis is one of the main causes of tooth loss in the adult human population [1]. The risk of edentulism and improper nutrition (due to the masticatory dysfunction) negatively affects the quality of life and self-esteem [5]. As a low-grade systemic inflammatory process, periodontitis can also influence many systemic disorders (i.e., atherosclerosis, hypertension, hyperglycemia, kidney disease, left ventricular hypertrophy, increased incidence of cardiovascular events) [27,28,29]. Periodontitis may have an impact on the risk of preterm birth and the low birth weight of newborns [30].

In the present study, an accepted experimental model of periodontitis developed by Novotny and Sanavi in 1983 was used [31]. The maxillary second molar was selected for the induction of periodontitis because only this rat molar has two interdental spaces [31]. Unlike in humans, in the rat’s maxilla, a large diastema separates the incisor from the first molar, so there is no mesial contact point and no interdental space between the incisor and the maxillary first molar.

In this model, as previously demonstrated, the development of periodontitis is accompanied by maxillary alveolar bone resorption in both the mesial and distal spaces [32], together with activation of the RANKL/RANK/OPG pathway [33]. Advanced inflammatory infiltration in the epithelium and connective tissue of the gingiva, as well as a reduction in the epithelial tissue, were also observed [34]. Ligation also resulted in inflammatory infiltration of various degrees in the periodontal ligament [35]. Subsequently, significant increases in the number of osteoclasts with high resorptive activity as well as osteoblasts were detected, resulting in the alveolar bone resorption accompanied by the formation of bone voids [33,34,35,36]. Numerous CD68-positive cells around the crest of the alveolar bone were observed [35].

Previous studies demonstrated the involvement of histamine H_2_ and H_4_ receptors in the development of experimental periodontitis [37,38,39]. The H_2_ receptor antagonist cimetidine reduces the number of inflammatory cells within the gingiva in these conditions [37]. It can also stimulate neutrophils present in the gingival fluid during the inflammatory process [38]. In addition, H_2_ antagonists have also been proven to influence B-cell and T-cell functions since the inhibition of IgG and IgM production caused by histamine could be reversed by cimetidine [38].

On the other hand, topical administration of histamine H_4_ receptor antagonist JNJ7777120 also reduced the inflammatory infiltration and hyperkeratosis in gingival tissue, and it decreased the presence of subepithelial inflammatory infiltrates [39].

Alveolar bone resorption induced by osteoclasts in periodontitis is modulated by the RANKL/RANK/OPG pathway [40]. RANKL acting via RANK is essential for osteoclast differentiation [8]. Studies by Hienz et al. demonstrate that the inflammatory process in periodontal tissues is accompanied by an increased RANKL/OPG ratio [14].

The activation of the RANKL/RANK/OPG pathway in experimental periodontitis was previously confirmed [15,41]. The use of cimetidine decreased the ratio of RANKL/OPG in the gingival connective tissue, and thus, resulted in reduced alveolar bone loss in these conditions [37].

Our studies demonstrate no influence of DL76 on RANK expression in the course of periodontitis. However, the expressions of epithelial RANKL and epithelial and stromal OPG were lower after DL76 treatment. The differences in the RANKL and OPG expressions support the finding of the effect of DL76 on the development of experimental periodontitis. These results are in line with the study presenting the anti-inflammatory action of curcumin in the same experimental model [42]. In contrast, the treatment with the cinnamic acid [43] and telmisartan [44] induced a decrease in the RANKL expression and the overexpression of OPG in a rat model of ligature-induced periodontitis. Despite these differences, the results of the present study proved the suppression of the RANKL/RANK/OPG pathway, since the expressions of both RANKL and OPG were decreased in the gingival tissues of DL76-treated animals compared to the control group.

A previous study proved that the H_1_ receptor antagonist pheniramine reduces alveolar bone loss induced by glucocorticoid administration in an experimental model of periodontitis in rats [45]. In addition, a study by Sun et al. confirmed that histamine H_1_ receptor antagonists prevent bone loss, increase bone density and its mineralization, and affect osteoblast differentiation [46].

On the other hand, the H_2_ receptor antagonists cimetidine and ranitidine were able to reduce the number of osteoclasts and decrease alveolar bone resorption in experimental periodontitis in rats [45,47], and they could inhibit alveolar bone loss caused by glucocorticoid administration [45]. Interestingly, simultaneous application of these compounds did not induce any cumulative protective effects. On the contrary, the effect was abolished [45]. Cimetidine was also proved to inhibit the inflammatory process with bone loss in a rabbit model of periodontitis [48]. In addition, the H_4_ receptor antagonists JNJ7777120 and JNJ10191584 were able to reduce the alveolar bone loss in experimental periodontitis in rats [39]. The mechanism may be related to H_4_ receptor-mediated stimulation of monocyte RANKL expression and osteoclast differentiation evoked by histamine, as demonstrated in patients with rheumatoid arthritis [49].

In the present study, we demonstrate DL76-mediated inhibition of bone resorption after the induction of periodontitis. The medians of the mesial and distal interdental spaces in DL_R_ were 3.56-fold and 10-fold lower, respectively, compared to S_R_.

DL76 was described for the first time in 2006 as an antagonist/inverse agonist of histamine H_3_ receptors [50]. However, later in vitro and in vivo studies, surprisingly, indicated its modulating effect on the course of the inflammatory process and the activity of cells involved in this reaction [51,52]. Our present results, demonstrating a reduction in the severity of experimental periodontitis, are in line with the studies by Grosicki et al. presenting a dose-dependent DL76-evoked inhibition of eosinophils adhering to endothelial cells [51] as well as by Mogilski et al. showing a reduced croton oil-induced ear edema and pruritus in CD-1 mice after DL76 treatment [52].

## 4. Limitations of the Study

Although our results clearly present DL76-induced inhibitory effects in a rat model of periodontitis, we are aware of the limitations of the work, especially concerning the mechanisms involved. Therefore, we suggest several possible mechanisms of DL76 action. Firstly, since histamine H_3_ receptors act as presynaptic auto/heteroreceptors within the central and peripheral nervous system [17], it could act as a modulator of the release of histamine and other neurotransmitters from presynaptic endings, and thus, influence neural regulation of the progression of the inflammatory process [24] and bone metabolism in the course of periodontitis [25,26]. Secondly, we cannot exclude the influence of DL76 on histamine H_4_ receptors; however, the compound has a low affinity for these receptors (hH4R Ki = 66 ± 36 microM) [53]. Thirdly, we hypothesize a possible mechanism of DL76 action via another type/s of receptors to which it may have the affinity. Finally, the non-specific effects of DL76 on immune cells, such as the modulation of intracellular enzyme activity and the influence on the synthesis of second messengers, cannot be excluded.

## 5. Materials and Methods

The experimental procedures were carried out in accordance with the Act on the Protection of Animals Used for Scientific or Educational Purposes of 15 January 2015. The research application was approved by the Local Ethics Committee, Katowice, Poland (Notification No 86/2015).

The studies were conducted in 24 mature male Wistar rats weighing 245–360 g, aged 6–8 weeks, purchased by the Centre for Experimental Medicine, Medical University of Silesia, Katowice, Poland. The animals were kept in separate stainless-steel, wire cages at a temperature of 22–24 °C, humidity 52–56% and 12 h light/dark cycle (rodent chow and tap water ad libitum) [54]. The animals were divided into two groups (each consisted of 12 individuals) [54]: the control saline-treated group (S), in which 0.9% NaCl (0.2 mL) was administered once daily, subcutaneously, for 28 days (from the day of ligating) and the experimental group (DL), in which DL76 (6 mg/kg dissolved in 0.2 ml of 0.9% NaCl) was administered once daily, subcutaneously, for 28 days (from the day of ligating). Each group was divided equally into two subgroups for the histopathological/immunohistochemical and radiological examinations, respectively. To avoid skin reactions, an abdominal body area with skin loose enough to allow volume expansion was selected for the subcutaneous administration. The temperature of the injected fluid was close to body temperature. The number of rats used in the study was the minimum necessary for statistical evaluation of the results, in accordance with the 3Rs guidelines for the humane treatment of animals. The studies were conducted in two rounds—the first one with the control, saline-treated group (S) and the second with the DL76-treated animals (DL76). The animals were observed during an acclimation period of 7 days, and only the rats without any symptoms of the disease were included in the study. General and local exclusion criteria were selected. The general pre-study exclusion criteria were clinical signs of pain and disease [55]. The local exclusion criteria before the study were absence of molars or absence of interdental spaces (large diastema separating maxillary second molars from first and/or third molars) and inflammatory changes occurring within the periodontal tissues. All animals without signs of general and/or local pre-study exclusion criteria were included in the 28-day experimental period. The general (death of the experimental animal, symptoms of any disease) and local (removal of the ligature) exclusion criteria within 28 days of the experimental period were selected.

Periodontitis was evoked by ligating (sterile, silk ligature, size 3–0) the neck of the second molar of the right maxilla (R) [31,32]. The procedure was performed under general anesthesia (10 mg/kg xylazine + 100 mg/kg of ketamine, intramuscularly). The second molar of the left maxilla (L) was treated as a control, where all the procedures, except ligating, were performed. After 4 weeks, under general anesthesia, the biological material was collected for testing, and the animals were euthanized.

### 5.1. Characteristics of the Inverse Agonist/Antagonist of the Histamine H_3_ Receptor, DL76

DL76 (1-[3-(4-*tert*-butylphenoxy)-propyl] piperidine) is a nonimidazole antagonist/inverse agonist of histamine H_3_ receptors. The compound has a solid form. Its melting point is 149–151 °C and its molecular weight is 365.48 [50,56]. After administration, it is rapidly distributed into all the organs of rats [57].

### 5.2. Immunohistochemical Analysis

The gingival tissue was preserved in 10% neutral buffered formalin [58]. For light microscope estimation of the inflammatory infiltration, the paraffin-embedded blocks were sectioned using rotary microtome and stained with hematoxylin and eosin.

Each evaluated specimen contained a fragment of mucosa covered with stratified squamous epithelium (orthokeratosis). The histopathological preparations were evaluated for the presence of inflammatory infiltrates [59].

For the immunohistochemical staining (IHC), paraffin blocks with sections of gingival tissue were cut on a rotary microtome. The 4 μm tissue sections were placed on the silicate glass surfaces of silanized slides and then deparaffinized with xylene, hydrated by passing the slides slowly through series of decreasing concentrations of alcohols and rinsed in distilled water. Then, the heat-induced antigen retrieval was carried out using Heat-Induced Epitope Retrieval Buffer at pH 6, lasting 20 min at 95 °C. Blocking of the endogenous peroxidase activity and the causes of non-specific binding of the antibodies to other proteins was performed by incubating the tissue sections firstly with 3% hydrogen peroxide and then with a protein block. The next stage of the IHC reaction was the incubation of the slides in a humidity chamber with the following primary antibodies: RANK (H-7: sc-374360, 1: 100) 4 °C, 16 h, RANKL (N-19: sc-7628, 1: 300) 4 °C, 16 h, OPG (N-20: sc-8468, 1: 200) 4 °C, 16 h.

After the incubation, the tissue sections were buffered three times in Tris-Buffered Saline (TBS) and then incubated for 10 min with the secondary antibody Primary Antibody Amplifier Quanto. In the subsequent stages, a 10 min incubation with a polymer HRP Polymer Quanto was performed, followed by visualization of the reaction product using 3-3′-diaminobenzidine chromogen DAB Quanto and contrasting by hematoxylin staining of the nuclei. Then, the preparations were dehydrated and sealed. The negative control for each antibody was obtained by conducting the above-mentioned procedure and skipping the primary antibodies. The IHC reactions were subjected to microscopic analysis. A positive result was determined on the basis of the cell location. Brown cytoplasmic reactions were considered to be positive.

To assess the presence of the RANK, RANKL and OPG in the gingiva epithelium and in the stroma, a 4-stage semi-quantitative method was used: grade 0—no reaction, grade 1—1–25% positive cells, grade 2—26–50% positive cells, grade 3—more than 50% positive cells [60]. The analysis was carried out separately for the stratified squamous epithelium and for the stroma. The histopathological preparations and IHC examinations were evaluated using a light microscope.

### 5.3. X-ray Pictures of the Rat Maxillas

X-ray pictures of the rat maxillas were taken using the Nanodor 2 X-ray unit set with an exposure time of 0.12 s. The lamp was set at 40 kV, 20 mAs.

### 5.4. Linear Measurement of Alveolar Bone Loss

The linear measurement of the alveolar bone loss was assessed using the RadiAnt Dicom viewer. The linear measurements (in mm) of the bone loss in the mesial and distal interdental spaces were produced from the cemento–enamel junction to the alveolar bone crest [31].

### 5.5. Description of the Applied Statistical Methods

Statistical analysis of the bone loss and IHC variables was performed using IBM SPSS Statistics 25 software.

### 5.6. Statistics concerning Bone Loss in Rat Maxillas

The Kruskal–Wallis test was used to check whether there were statistically significant differences between the examined groups. When statistically significant differences occurred, the Games–Howell post hoc test was used to assess in which groups there were statistically significant differences. The mean, standard deviation, median, minimum and maximum were estimated. A *p* value < 0.05 was considered to be statistically significant.

### 5.7. Statistics concerning IHC Variables

The Kruskal–Wallis test was used to check whether there were statistically significant differences between the examined groups. When statistically significant differences occurred, the adequate post hoc test was used to assess in which groups there were statistically significant differences. The Mann–Whitney U test was used to compare the differences between two groups. The Chi-square test was used to determine whether there was a significant relationship between two nominal variables [61,62]. The mean, standard deviation, median, minimum and maximum were estimated. A *p* value < 0.05 was considered to be statistically significant.

## 6. Conclusions

In conclusion, the H_3_ receptor antagonist/inverse agonist DL76 is able to inhibit the development of experimental periodontitis in rats by decreasing the activity of the RANKL/RANK/OPG pathway in the gingival tissues and reducing the resorption of the maxillary alveolar processes.

## Figures and Tables

**Table 1:** Descriptive statistics of the percentage variables of the epithelial RANKL, RANK and OPG expression in the S_L_ (*n* = 6), S_R_ (*n* = 6)_,_ DL_L_ (*n* = 6) and DL_R_ (*n* = 6) groups.

Variable		M	Me	SD	Min	Max	Statistical Test Result *
RANK-e	S_L_	55%	67.5%	28.64%	10%	80%	χ^2^(3) = 2.07; *p* = 0.56
	S_R_	71.67%	72.5%	6.83%	60%	80%	
	DL_L_	60.83%	65%	13.57%	40%	75%	
	DL_R_	66.67%	70%	11.69%	50%	80%	
RANKL-e	S_L_	0.83%	0%	2.04%	0%	5%	χ^2^(3) = 9.3; *p* = 0.03
	S_R_	15%	20%	12.25%	0%	25%	
	DL_L_	0%	0%	0%	0%	0%	
	DL_R_	0.83%	0%	2.04%	0%	5%	
OPG-e	S_L_	0%	0%	0%	0%	0%	χ^2^(3) = 10.77; *p* = 0.01
	S_R_	5%	5%	4.47%	0%	10%	
	DL_L_	0%	0%	0%	0%	0%	
	DL_R_	0.83%	0%	2.04%	0%	5%	

e—epithelium; * Kruskal–Wallis test.

**Table 2:** The expression of RANK, RANKL and OPG in the epithelium and stroma in the studied groups.

Variable		*n* (%)				Statistical Test Result *
		Lack of Expression	Low Expression	Average Expression	High Expression	
RANK-e	S_L_	0 (0%)	1 (16,7%)	1 (16.7%)	4 (66.6%)	χ^2^(6) = 5.58; *p* = 0.47
	S_R_	0 (0%)	0 (0%)	0 (0%)	6 (100%)	
	DL_L_	0 (0%)	0 (0%)	2 (33.3%)	4 (66.7%)	
	DL_R_	0 (0%)	0 (0%)	1 (16.7%)	5 (83.3%)	
RANK-s	S_L_	0 (0%)	6 (100%)	0 (0%)	0 (0%)	χ^2^(3) = 9.78; *p* = 0.02
	S_R_	0 (0%)	6 (100%)	0 (0%)	0 (0%)	
	DL_L_	4 (66.7%)	2 (33.3%)	0 (0%)	0 (0%)	
	DL_R_	2 (33.3%)	4 (66.7%)	0 (0%)	0 (0%)	
RANKL-e	S_L_	5 (83.3%)	1 (16.7%)	0 (0%)	0 (0%)	χ^2^(3) = 8; *p* = 0.046
	S_R_	2 (33.3%)	4 (66.7%)	0 (0%)	0 (0%)	
	DL_L_	6 (100%)	0 (0%)	0 (0%)	0 (0%)	
	DL_R_	5 (83.3%)	1 (16.7%)	0 (0%)	0 (0%)	
RANKL-s	S_L_	5 (83.3%)	1 (16.7)	0 (0%)	0 (0%)	χ^2^(3) = 2.18; *p* = 0.54
	S_R_	5 (83.3%)	1 (16.7)	0 (0%)	0 (0%)	
	DL_L_	6 (100%)	0 (0%)	0 (0%)	0 (0%)	
	DL_R_	6 (100%)	0 (0%)	0 (0%)	0 (0%)	
OPG-e	S_L_	6 (100%)	0 (0%)	0 (0%)	0 (0%)	χ^2^(3) = 10.86; *p* = 0.01
	S_R_	2 (33.3%)	4 (66.7%)	0 (0%)	0 (0%)	
	DL_L_	6 (100%)	0 (0%)	0 (0%)	0 (0%)	
	DL_R_	5 (83.3%)	1 (16.7%)	0 (0%)	0 (0%)	
OPG-s	S_L_	6 (100%)	0 (0%)	0 (0%)	0 (0%)	χ^2^(3) = 10.29; *p* = 0.02
	S_R_	3 (50%)	3 (50%)	0 (0%)	0 (0%)	
	DL_L_	6 (100%)	0 (0%)	0 (0%)	0 (0%)	
	DL_R_	6 (100%)	0 (0%)	0 (0%)	0 (0%)	

e—epithelium; s—stroma; * Chi-square test.

**Table 3:** Statistical analysis of the mesial and distal interdental spaces (in mm) in the studied groups: S_L_ (*n* = 6), S_R_ (*n* = 6), DL_L_ (*n* = 6), DL_R_ (*n* = 6).

Variable		M.	Me	SD	Min	Max	Statistical Test Result *
Mesial space	S_L_	0.39	0.41	0.09	0.25	0.51	χ2 (3) = 20.25; *p* < 0.001
	S_R_	0.79	0.82	0.17	0.54	0.98	
	DL_L_	0	0	0	0	0	
	DL_R_	0.18	0.23	0.16	0	0.33	
Distal space	S_L_	0.37	0.15	0.14	0.25	0.64	χ2 (3) = 20.85; *p* < 0.001
	S_R_	0.84	0.8	0.2	0.58	1.13	
	DL_L_	0	0	0	0	0	
	DL_R_	0.09	0.08	0.1	0	0.2	

* Kruskal–Wallis test.

## Data Availability

The datasets presented in this article are not available because of an ongoing study.
